# Broadening horizons: the case for capturing function and the role of health informatics in its use

**DOI:** 10.1186/s12889-019-7630-3

**Published:** 2019-10-15

**Authors:** Denis Newman-Griffis, Julia Porcino, Ayah Zirikly, Thanh Thieu, Jonathan Camacho Maldonado, Pei-Shu Ho, Min Ding, Leighton Chan, Elizabeth Rasch

**Affiliations:** 1 Rehabilitation Medicine Department, National Institutes of Health, Mark O. Hatfield Clinical Research Center, 6707 Democracy Boulevard, Suite 856, MSC 5493, Bethesda, MD 20892 USA; 2 Department of Computer Science and Engineering, The Ohio State University, 2015 Neil Avenue, DL 395, Columbus, OH 43210 USA; 3 Department of Computer Science, Oklahoma State University, 116-A MSCS, Stillwater, OK 74078 USA; 4 Information Technology Laboratory, National Institute of Standards and Technology, 100 Bureau Drive, Gaithersburg, MD 20899 USA

**Keywords:** Disability evaluation, Electronic health records, Health informatics, Clinical informatics, Public health informatics, Natural language processing

## Abstract

**Background:**

Human activity and the interaction between health conditions and activity is a critical part of understanding the overall function of individuals. The World Health Organization’s International Classification of Functioning, Disability and Health (ICF) models function as all aspects of an individual’s interaction with the world, including organismal concepts such as individual body structures, functions, and pathologies, as well as the outcomes of the individual’s interaction with their environment, referred to as activity and participation. Function, particularly activity and participation outcomes, is an important indicator of health at both the level of an individual and the population level, as it is highly correlated with quality of life and a critical component of identifying resource needs. Since it reflects the cumulative impact of health conditions on individuals and is not disease specific, its use as a health indicator helps to address major barriers to holistic, patient-centered care that result from multiple, and often competing, disease specific interventions. While the need for better information on function has been widely endorsed, this has not translated into its routine incorporation into modern health systems.

**Purpose:**

We present the importance of capturing information on activity as a core component of modern health systems and identify specific steps and analytic methods that can be used to make it more available to utilize in improving patient care. We identify challenges in the use of activity and participation information, such as a lack of consistent documentation and diversity of data specificity and representation across providers, health systems, and national surveys. We describe how activity and participation information can be more effectively captured, and how health informatics methodologies, including natural language processing (NLP), can enable automatically locating, extracting, and organizing this information on a large scale, supporting standardization and utilization with minimal additional provider burden. We examine the analytic requirements and potential challenges of capturing this information with informatics, and describe how data-driven techniques can combine with common standards and documentation practices to make activity and participation information standardized and accessible for improving patient care.

**Recommendations:**

We recommend four specific actions to improve the capture and analysis of activity and participation information throughout the continuum of care: (1) make activity and participation annotation standards and datasets available to the broader research community; (2) define common research problems in automatically processing activity and participation information; (3) develop robust, machine-readable ontologies for function that describe the components of activity and participation information and their relationships; and (4) establish standards for how and when to document activity and participation status during clinical encounters. We further provide specific short-term goals to make significant progress in each of these areas within a reasonable time frame.

## Background

The way in which we learn about our world as individuals and how we willfully act within it is fundamental to human existence. Human activity, and the impact of health conditions on it, is an important component of contemporary conceptualizations of health. This article is the product of a collaborative effort between an interdisciplinary group of health professionals and scientists to summarize the importance of capturing information on activity in health systems, and to identify analytic tools and techniques that can support effective utilization of this information for improving patient care. We draw on particularly relevant references in the salient fields to highlight the concepts, techniques, and evidence for a broader incorporation of activity information into healthcare and data analytics. We first describe conceptualizations of human activity and its role in models of health and disability, and existing methods and applications for measurements of activity. We then identify current information gaps regarding activity, along with methods for improving the rate and quality of capture of activity information and analyzing it to inform care decisions. Finally, we suggest four specific actions that can be taken towards more effective use of activity information in health systems, and identify practical short-term goals to make meaningful progress towards each.

### Activity and disability

In sociology, action theory describes human activity, and its purposeful nature, in the context of environments and societies in which activities take place. Although first described in 1937 [1], the concept of human action has more recently been applied to the fields of medicine and health sciences to characterize the consequences of health conditions as an important and meaningful indicator of health. This concept is reflected in contemporary models of disability, for instance, where disability is conceptualized as the outcome of the interaction between the capabilities of individuals and the demands of environments with which individuals interact. The premise that disability reflects how people function given a particular context was articulated by Saad Nagi in the early 1960’s [2] and formed the basis for every contemporary model of disability that followed. Now codified in the World Health Organization’s (WHO) International Classification of Functioning, Disability, and Health (ICF) [3] and adopted internationally, human action is embodied in the domain of activity and participation, where activity represents the execution of an action by an individual and participation represents actions through involvement in life situations. Actions, which take place at the level of the individual, are distinguished from organ or organ system function (ICF body structures/functions), or cellular/tissue function (ICF health conditions).

### What is function?

**Fig. 1:**
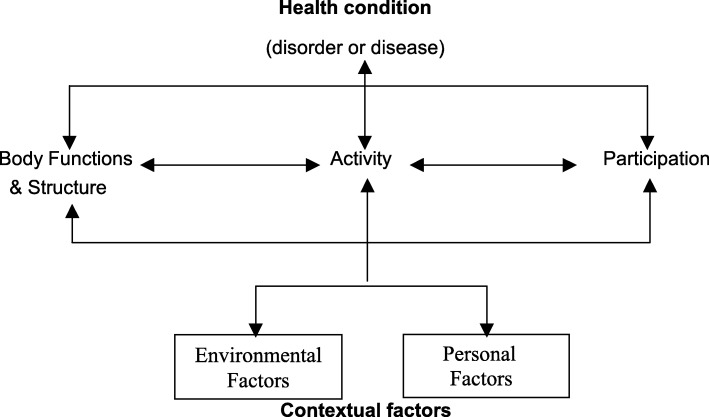
Diagram of the International Classification of Functioning, Disability and Health (ICF) model of function. Reproduced by permission of World Health Organization (WHO), from ICF [3], p18

Human function can be broadly conceptualized as a continuum from body structures and functions to outcomes of interactions between individuals and their environments [4, 5], and has been argued to reflect “the lived experience of health” [6, 7]. The ICF defines function as an umbrella term encompassing all aspects of the interaction “between an individual (with a health condition) and that individual’s contextual factors (environmental and personal factors)” [4]. Within the ICF model, function is broken down into several components, illustrated in Fig. 1. This model encompasses all aspects of an individual’s interaction with the world, including organismal concepts such as individual body functions/structures and pathologies, as well as activity and participation, and all the environmental factors that affect these interactions. Importantly, activity and participation reflect volitional actions that take place at the level of the whole person, such as walking, communicating, applying knowledge, etc., which take place in, and are influenced by, a life situation or social context. For the purposes of this article, we operationalize the term “function” at this whole person level, and refer primarily to “activity and participation” in detailed discussion.

### Why are activity and participation important health indicators?

At both the individual and population levels, the ability of people to engage in activities and their participation in social roles shapes the need for resources and the associated response from national agencies, health systems, home and community-based organizations, and other support entities [8]. One timely example of the need for information about activities and participation on a global scale is a consequence of the dramatic shift in the world’s demographic profile due to population aging. Among figures that the United Nations (UN) calculates in relationship to population ageing is the support ratio, which is the number of workers per retiree. By 2050, 36 countries, including the U.S., are expected to have support ratios below 2 [9], meaning that there will be fewer than 2 working persons to support each person over the age of 60. Ultimately, an individual’s independence and ability to participate in meaningful life activities (i.e., quality of life) will heavily influence resource needs [10] and, at the population level, will have an overwhelming impact on national public health, pensions, and social programs serving the elderly. As noted in the WHO World Report on Ageing and Health, complex health states resulting from the coexistence of multiple chronic conditions (which can exist at any age) are not adequately represented by identifying or treating one disease at a time. As a result, there is a need for measures that are more meaningful to individuals [5].

The need for better information on activity and participation at the individual level has also been widely endorsed [11, 12]. Activity and participation reflect the cumulative outcome of disease burden, i.e. multimorbidity. In the U.S., it has been reported that over half of working age adults experience one or more chronic conditions [13]. It is well established that there is a strong and consistent association between a greater number of chronic conditions and the existence and severity of limitations in activities and participation [14, 15]. Thus, the effect of multiple chronic conditions on the lives of individuals is realized in their overall function [6, 7]. Since function reflects, among other factors, the cumulative impact of health conditions on the person, and is not disease specific [16], its use as a health indicator helps to address major barriers to holistic, patient-centered care, such as fragmentation in care resulting from multiple and often competing disease-specific interventions [17].

In clinical settings, the inclusion of information on activity and participation in case mix calculations has been shown to improve the prediction of patient needs and resource use [8]. Evidence suggests that in cases of multi-morbidity, reducing the complexity of an individual’s overall health state to approaches focusing on each disease individually fails to provide adequate care for this growing segment of the global population [18]. Viewing the outcome of these complexities in the form of whole person function, i.e., activity and participation, is therefore likely to clarify approaches to intervention [8, 10]. Function reflects a health continuum and thus is more comprehensive in its characterization of health than other endpoints like morbidity or mortality [17]. Indicators of function are strongly predictive of mortality [19] but have the additional advantage of being more proximal health indicators, permitting earlier and potentially more effective interventions [10, 20]. Simple and objective tests of physical performance have been included as biomarkers in studies of ageing, outperforming more traditional impairment measures in models predicting mortality [20]. Markers of frailty that include physical function have been associated with employment difficulties in late middle age [21]. In addition to predicting mortality, indicators of physical function have been shown to predict other important and more immediate outcomes such as subsequent disability [22] and dementia [23] among older adults. In the context of population ageing, the prevalence of multi-morbidity within populations and within individuals will have associated consequences in function. Thus, information about function at both the individual and population level is critical for the design of healthcare systems, home and community-based supports, and for resource allocation.

### How have activity and participation been measured?

Models of function have historically been developed in the context of discussing disability, which is often described in terms of limitations in function [2, 24, 25]. However, these are *conceptual* models, describing the broad components that contribute to function, and have proven difficult to translate to *data* models that can capture specific aspects of function in context and how they relate to one another. Even the ICF, the most detailed framework developed for function, does not formally describe the relationships between different structures, activities, and environmental factors. Thus, how best to measure function, and particularly activity and participation, remains an open question despite international efforts [26, 27]. Many of the existing measurements are at the population level, in the form of national survey questions (see [26] for a detailed review of many such survey instruments). While these are relatively easy to administer with high coverage, they are necessarily limited in detail, in order to minimize respondent burden, and are unable to capture the individual perspective. Some efforts have been made to systematically capture information on activities of daily living (ADLs) in individual healthcare encounters; however, these have been captured relatively rarely and only present one small piece of the overall picture of activity and participation [27, 28]. Notably, information about the *environment* in which an individual functions is rarely captured under either approach, despite being central to concepts of function and disability. This continuing debate and development of instruments to measure function means that even where measurements of activity and/or participation are captured, they cannot easily be recognized as such or mapped to standardized vocabularies and data models for analysis.

### Definitions and examples of terms

One effect of the malleable definitions of function and its measurement is that language used for these concepts varies widely, particularly between different scientific fields. For clarity, we define our key terms here, and provide examples of each.

- **Function** – “a dynamic interaction between a person’s health condition, environmental factors, and personal factors” [3]. This is an umbrella term including cellular and tissue function, organ and body structure function, and whole person function.
- **Activity and participation** – the outcome of the interaction between an individual (with some health condition) and their environment, including specific activities and participation, as well as personal contextual factors; also referred to as whole person function. This encompasses basic willful actions, specific tasks, organized activities, and role participation [26, 29]. Examples include walking (including the environment being walked on, anything used to assist in performing the activity, etc), taking public transportation (which combines walking with other activities such as identifying a destination, sitting, etc), or participating in work.
- **Activity report** – a recorded observation of activity and/or participation, which identifies relevant components of a specific activity or participation outcome and records them in structured or unstructured data. Examples include, “Patient walked one lap in the hallway,” or “Sue reports to work every day at 9 and works with no limitations until 5pm.” Prior work has referred to information samples of this type variously as “functioning information” [30], “functional status terms” [31], “functional status information” [32], “functional health status” [33], and other terms. However, prior studies have not specifically distinguished information about activity and participation from information about other elements of function; thus, we adopt the term “activity report” to clearly distinguish activity and participation information from other types of health information.

## The information gap: What’s missing?

While information on pathology, and even impairments of individual body functions, has been captured at a high rate for use in many modern health systems [34], information on activity and participation is captured relatively rarely and remains difficult to use effectively [7, 35]. In order to utilize data on activity and participation, i.e., activity reports, the healthcare field has two primary needs: (1) standardized procedures and tools for capturing activity reports routinely and quickly (both in and out of the clinic), and (2) methods for analyzing activity reports to support evidence-based decision making. We suggest approaches towards meeting both of these needs, and provide four concrete calls to action, with example short term goals for each, to improve both the availability and the utility of activity and participation information for modern health systems.

### How can information on activity and participation be captured?

At the population level, most countries collect basic information on function via national censuses and surveys [36], but this information is rarely captured in sufficient detail or frequency to have an impact on healthcare systems [7]. Thus, national surveys cannot be responsive to information needs in real time. At the individual level, some self-administered surveys for measuring specific aspects of functional status have been developed [37], and social media technologies have been shown to passively capture some information about individual function [31]; wearable devices are also an emerging technology for capturing individuals’ activity-related information. However, these tools are, at least currently, difficult to standardize and apply to reliably capture information on activity and participation at scale. Health systems, which many individuals encounter fairly regularly, offer another logical source for capturing information about activity and participation, which can be combined with other sources for a fuller picture of individual function. While some information about activity and participation is already collected during healthcare encounters, there remains significant variability in terms of how often and on whom it is collected, as well as what information is captured [7, 17, 20, 35]. In addition to objective observations of activity and participation, expanded documentation of activity reports in health records can also capture self-reported data, which complements clinical assessments [28, 38].

The current scarcity of activity reports at the individual level, recorded via diverse modalities, instruments, and language, presents challenges for their use in decision making. Firstly, to support evidence-based decision making in health systems, health information must be standardized and interoperable to optimize its potential usefulness [17]. Usefulness, in turn, can only be achieved when raw data are translated into knowledge that can change practice, requiring analytics. An extraordinary volume of data is generated in health systems [39], and many of these data may include errors that impact analytics [40, 41]. Coordination with data from surveys, self-reported tools, and other media can improve accuracy, but increases the volume of data that must be processed. Thus, concerted efforts are needed to tap into the potential of these sources of information on activity and participation. A data-driven approach leveraging current techniques in health informatics to extract information about function, in particular activity and participation, is needed and represents an effort that requires the involvement and coordination of many entities [5].

### How can information on activity and participation be analyzed?

The field of health informatics involves the use of health-related data for scientific inquiry and discovery and for decision making in healthcare and government [42]. This definition encompasses a wide variety of analytic methods, which can be broadly separated into analyses of structured data (i.e., data fields such as vital signs, demographics, lab readings, etc) and unstructured data (e.g., free-text health records or medical images). Analysis of structured data has proven invaluable in advances in medical informatics and public health, such as monitoring cancer incidence and treatment at a population level [34], predicting the need for specific interventions in individual breast cancer treatment [43], cohort identification in Nordic countries [44], and many others [45, 46]. In the area of functional status measures and its correlation to mortality risk, factors such as age, gender, and some ADL information have been used to predict 2-year mortality [47]. However, a lack of standardized data models means that activity reports are difficult to capture in structured form. Even where some simpler aspects such as ADLs are captured in health records, they are difficult to correlate across samples [35]; existing structured judgments also often lack the granularity to capture functional limitations informatively [48]. Ongoing development of standards for recording information relevant to activity, such as physical therapy outcomes, offers one way to improve capture of structured data for analysis [49]. Further, imaging techniques are growing as an area of assessing impairments and associated functional limitations [50, 51], although such techniques impose high provider burden. Thus, we focus our discussion on unstructured text—particularly in health data—where activity reports have historically been captured [28, 48], and which offers flexilibity to capture relevant details such as environmental or personal factors. While this flexibility can contribute both to provider burden in writing documentation and analytic burden in extracting useful information from it [52, 53], technologies such as speech recognition and natural language processing (NLP) can be used to reduce this burden while enabling automatic extraction, organization, and summarization of relevant information [53–55].

### How has NLP been used in clinical care and research?

Natural Language Processing (NLP) is a broad field of research that has been used for a variety of purposes in processing health-related text data. The most common application of NLP for health has been automatically extracting and recognizing health-related information in text [56–58], such as symptoms, procedures, and diseases [59–61], medications [62, 63] health events [64, 65], and patient characteristics [66], among other examples. Many advances in NLP for health have been enabled through shared tasks [67], which engage a wider research community to solve a specific research problem such as detecting smoking status [68] or heart risk factors [69]. NLP has a long history of research and operational use in clinical informatics [70], and has proven especially helpful for several tasks that are difficult or expensive for humans to complete, such as detecting rates of patient readmission to different facilities [71]. NLP methods have also been incorporated operationally in diverse decision support systems including modeling disease progression, identifying cancer-related information in pathology reports, and risk assessment tools [72, 73].

While NLP for healthcare applications has historically focused on diagnostic information such as diseases, symptoms, medications, and procedures, more recent research is expanding both within and outside the clinic to consider contextual factors and other data sources. For example, homelessness is an important social indicator of health that can be extracted from the text of clinical encounters [74, 75]. NLP techniques have also been instrumental in leveraging pervasive social media data for diverse applications, from detecting adverse drug reactions to epidemiological surveillance [72]. Social media data have been particularly transformative for monitoring and analyzing mental health, a critical component of function. For instance, NLP techniques have been used to assist moderators of online forums by automatically flagging posts suggesting a mental health crisis—such as suicide risk—for immediate human intervention [76]. Current efforts are also being put into creating datasets that would further application of NLP techniques in this domain [77, 78].

### How has unstructured activity and participation information been analyzed?

Structured data about activities, participation, and associated limitations are central to disability research, assistive technology development, and many other fields. These data can be gathered from national surveys [79, 80], obtained via specialized research instruments [81], or modeled from available clinical information [82], although use of this information in healthcare delivery remains relatively limited [83]. Analyzing unstructured text information about activity and participation, however, along with associated environmental and personal factors, is an emerging area of interest in health informatics research. Recent work has included collecting self-reported function terms by manually reviewing clinical documents and online forums [31], and identifying groups of phrases describing various aspects of function via clinical chart review [33]; notably, the majority of these terms were not found in established terminological resources like the Unified Medical Language System (UMLS) [84]. To address this issue of coverage, some researchers interested in activity and participation have utilized application-specific vocabularies compiled by clinical staff. Such handcrafted approaches have been successful in various applications, including automatically assigning some ICF codes in discharge summaries [85], using ICF codes for information retrieval [86], and predicting patients’ rehospitalization risk [87]. Other work has avoided the coverage issue by using vocabulary-agnostic methods that are targeted to specific types of activity reports [88]. Additionally, activity and participation information has been used in the extraction and modeling of other functional outcomes, such as frailty or grave illnesses, from clinical text [89–91]. These studies represent significant initial efforts in analyzing activity and participation information with NLP, but the lack of systematic alignment with an overall conceptual framework for activity and participation and lack of shared definitions of the analytic tasks pose challenges for synthesizing and building on these efforts.

### What is needed to improve analysis of activity and participation information?

While activity reports may not yet be commonplace or a robust part of medical records, important information on activity and participation is currently being recorded, and is most often located in the free text portions of clinical notes. Thus, we focus on NLP as a critical tool for capturing this information for use and analysis. NLP, like other techniques used in health informatics, is a complex field that relies on a multitude of resources to achieve optimal performance. In the following sections, we walk through several factors in effective informatics, what is needed to support them, and the particular challenges of supporting these needs in the context of activity and participation information analysis. These points are also summarized in Table 1.

**Table 1:** Four approaches to addressing the information gap on activity and participation

Approach:	Common datasets for research	Shared understanding of analytic tasks	Expert knowledge of activity and participation	Records of activity and participation
Analytic Needs:	• Volume: sufficient data to support modern methods of analysis. • Representation: data must be widely representative. • Annotation: gold standard descriptions of activity reports for benchmarking and comparison.	• Problem definitions: common definitions of analytic tasks and evaluation. • Problem sharing: information exchange in the community. • Interdisciplinary collaboration: input from clinical and analytic stakeholders.	• Standardized information structure: clear standards of information components and their relationships. • Robust sources of information: capture variation and common usage of language and data.	• Recorded observations: activity reports explicitly recorded during patient encounters.
Challenges for Activity and Participation:	• Records from general encounters often have few activity reports. • Activity reports are expressed in diverse language and in varying levels of detail. • No common datasets with activity and participation information available for community research.	• Prior NLP work on activity and participation information has been highly specific and does not generalize easily. • Requires both data science and clinical expertise to effectively adapt existing methods to data that contain activity reports.	• Existing resources lack sufficient structure to accurately represent activity and participation information in practice. • Current vocabularies have poor coverage of activity and participation concepts and terms.	• Multiple competing standards exist for documenting information in rehabilitation medicine. • Standards are not widely adopted outside of rehab for standard clinical care.
Action:	• Develop and publish standards for annotating activity reports. • Develop resources for research that can be shared through regulatory frameworks.	• Identify and define common research problems and applications for processing activity reports.	• Develop a clinically-informed ontology for activity and participation information, along with representative terminologies from multiple sources.	• Establish common standards for observing and documenting activity reports in patient encounters.
Short-term Goals:	• Develop and publish annotation schema for 1–2 specific aspects of activity and participation. Make small sets of annotated data available through existing data sharing mechanisms.	• Establish shared tasks for extracting particular activity reports from an annotated dataset.	• Develop mappings across existing conceptual frameworks, such as ICF and SNOMED.	• Identify minimal interventions that can capture high-impact activity and participation status.

### What data are needed for successful informatics?

Much of the potential of health informatics is predicated on the availability of data. To develop and evaluate informatics methods for activity and participation, it is necessary to have data that have been annotated, or marked by experts as to what relevant information it contains and where that information can be found. Annotation serves two primary roles in informatics: to tell analysts and machine learning systems what specific information to focus on; and to serve as a gold standard for evaluating proposed automated methods and supporting benchmarking and comparison within a broader research community.

Examples of annotations for activity and participation information might include highlighting descriptions of specific actions (e.g., walking, climbing, shopping, cleaning) or life situations in free text, or even what type of clinical evaluation is being described. Annotating such information requires both identifying and standardizing the components of activity reports in clinical records. Function is defined within the ICF as the outcome of the interaction of individuals with various contextual factors, which means that descriptions of activity and participation tend to be complex and rely on multiple pieces of evidence. For example, a therapist might observe that a patient is able to walk with a rolling walker for 300 ft. While the activity report that needs to be captured is focused on the action (“walk”), this information is contextualized by other factors such as the assistive device (“rolling walker”), and these relationships must be captured in annotation as well.

In addition to annotating data, it is important to devote research and administrative efforts to collecting and sharing large volumes of data that represent activity and participation information. Many recent advances in statistical methods for NLP, particularly deep learning technologies, have relied on the availability of thousands or millions of documents [92], but virtually no documents with activity and participation information are available to the broader research community at present. Semantic approaches leveraging expert knowledge have been used to great effect in low-data settings in the past [93]; however, such methods have typically relied on robust standardized resources that are lacking for activity and participation, emphasizing the value of statistical learning from large datasets.

In medical data, which often contains protected health information (PHI), there are two main strategies for collecting such datasets. First, research groups within a single institution or collaboration may collect private data under an IRB-approved protocol. These data may be re-used or shared after the initial study via mechanisms such as protocol amendments, designing new protocols, and developing business or data use agreements. While these tend to be limited to specific named parties included in the protocol or legal agreements, and may involve lengthy approval processes, such mechanisms have been effectively used for a large variety of data sharing scenarios in health research [94]. A second strategy is to curate de-identified datasets that remove PHI and are then made more widely available while taking appropriate precautions for data stewardship. This is not a simple task: though de-identification can be performed without significantly reducing relevant clinical information [95], it is by no means a perfect process [96, 97], and defining what qualifies as de-identified requires agreement between all relevant stakeholders, such as IRBs, privacy offices, government entities, and most certainly patients. De-identified datasets are thus rare, but have an outsize impact in supporting rapid and effective research within a whole community. Under any chosen mechanism, sharable datasets of activity reports will contribute significantly to informatics research and applications using activity and participation information.

### How do we make use of these data?

Applying informatic methods to use activity and participation information in clinical and administrative practice requires addressing a wide variety of analytic challenges. One challenge is that many specific analytic tasks do not clearly correspond to existing informatics research problems. For example, activity reports, such as “walks without gait aid 50 feet in hallway”, involve the interaction of several concepts. Recognizing and extracting such reports from text requires both identifying the component concepts (e.g., the action “walks”, environmental factors “in hallway” and “without gait aid”, and the specific distance “50 ft”) and linking them together. Walking in an indoor hallway is significantly different from walking across rough terrain outside; connecting these elements is necessary to extract the atomic outcome being recorded. This task is further complicated when multiple outcomes are described in a single report; for example, “ambulate in the hallway and stairs” refers both to walking and to climbing (two distinct activities in the ICF). Thus, modeling the complex semantics of activity reports may involve combining multiple existing research problems, such as named entity recognition, syntactic dependency parsing, and even conceptual inference.

Even well-studied problems such as information retrieval or relation extraction can face new challenges for activity and participation information. For example, some patient records, such as History and Physical Examinations, often contain only a few sentences describing physical and mental function among a much larger concentration of diagnostic history, past procedures, etc. For a healthcare provider or administrator attempting to locate activity and participation information about a patient, such as a physical therapist tracking activity history or an analyst surveying inpatient functional outcomes, it is therefore necessary to pinpoint which sections or paragraphs of a long document include important information to review. Furthermore, such users must be able to quickly access and intuitively organize patient records from a variety of disciplines. These applications encompass diverse NLP tasks, including information extraction and retrieval, for identifying and organizing activity and participation information in the medical record; knowledge representation, for capturing clinically-informed relationships between activity and participation concepts; and determining the relevance of documents with respect to particular criteria, such as potential limitations in function. As with all complex tasks and modern problem solving approaches, addressing these issues for practical care will require interdisciplinary collaboration between clinical or domain experts, knowledge representation specialists, and informaticians at all stages of the analytic process, from defining goals to practical implementation in healthcare systems.

### What resources do we need?

Beyond the quantity and quality of available data, many successful clinical applications of NLP have been enabled by robust medical knowledge sources. These sources are referred to by various names, including (but not limited to) taxonomies, terminologies, and ontologies. These terms are used inconsistently in the literature, so we define each of them for this article as follows. Terminologies capture the diverse names used to refer to biomedical concepts, such as diseases, substances, measurements, etc., and are intended to both catalogue distinct concepts and provide a more or less comprehensive reference for the ways these concepts can be referred to. Biomedical terminologies often include elements of domain-specific ontology in their structure, which describe invariant classes of concept, such as diseases, symptoms, biological processes, functions, etc. Ontology also describes relations that hold universally between these classes: for example, that convulsions are a symptom of seizure [98]. Many terminologies have been developed as formalized coding systems, and can be referred to as classifications or taxonomies; the International Classification of Diseases (ICD), another WHO reference classification, being a salient example. As a result, the organization of many terminologies distinguishes not only between ontologically different classes (e.g., febrile vs afebrile seizure), but also epistemologically distinct observations (e.g., tuberculosis identified via microscopy or bacterial culture) [98]. Both types have been critical components of many successes in health informatics [45, 99].

However, comparable knowledge sources are few and far between for non-medical aspects of function. The ICF, originally developed in 1980 as the International Classification of Impairments, Disabilities, and Handicaps (ICIDH) and revised in 2001 to better model environmental aspects of function [100], is a conceptual terminology that was designed to provide a common language for a wide variety of administrative and policy needs such as reporting, service coordination, and policy development [4]. Though the ICF has been integrated into the UMLS, and some efforts have been made to map it to other ontological resources [101], comprehensive coverage of practical vocabulary has never been its intent, and mappings to other well-developed terminologies such as SNOMED CT or LOINC are minimal. As a result, its coverage and granularity for coding practical information on activity and participation has been shown to lag behind higher-coverage medical terminologies [102]. Additionally, the distinctions it draws do not necessarily reflect a clinically-based organization of knowledge. As a practical example, the mobility-related action of *walking* is not linked within the ICF to terms commonly used in practice, such as *ambulation*. A recent review found several other criticisms of the organization of the ICF, such as its emphasis of the health condition component, the ambiguity of concepts, and its “lack of a clear ontological structure” [103]. Some of these criticisms may be related to the lack of revisions to the ICF over the years. While the WHO publishes updates to the language of the ICF each year, it has never been revised, unlike the ICD, which is currently under its 11th revision. Thus, while the ICF has been hailed as the “best prospect for an internationally recognized, sufficiently complete and powerful information reference for the documentation of functioning information” [17], and it has the potential to be effectively combined with other vocabularies for coding purposes [104], a number of practical shortcomings make it difficult to utilize for successful NLP methods relying on dictionary definitions or common patterns in order to extract activity and participation information.

## A call to action

Incorporating information on activity and participation into the operation of health systems is not a simple task, and fully utilizing activity and participation status to improve the quality of life of populations and individuals will require concerted long-term efforts. In the following sections, we describe four major components of this overall goal. These approaches are highly inter-related, but reflect distinct steps to be taken by the medical and research communities to enable greater capture and utilization of activity reports. While these steps are complex and may require coordination between international entities, we have identified short-term goals that can achieve significant initial progress within a reasonable time frame.

### Action 1: Develop annotation standards and data

In order to understand how to process activity and participation information as it is currently documented, it is necessary to **develop and publish standards for annotating activity reports in structured and unstructured data, and develop data resources for research that can be shared through regulatory frameworks.** Preliminary investigations into the variety of ways in which activity reports are documented in various text sources can lay the groundwork for this effort, but published annotation standards establish a common base for communication and comparison within the research community. Development of sharable datasets regarding individuals’ health data faces significant challenges in data privacy and interoperability, as well as a lack of robust legal frameworks or incentives for development [105]. However, there are well-developed risk-tolerant mechanisms for data exchange, including IRB procedures, data use agreements, and business agreements [106], and when such mechanisms are used, sharable datasets contribute significantly to rapid advancement of research. For example, the MIMIC Critical Care database is a de-identified dataset made available through a signed data use agreement that, through active maintenance, has expanded to include over 2 million text documents in addition to lab readings, vital signs, etc. [107]. MIMIC has been invaluable for clinical informatics and NLP research into extracting diagnoses, symptoms, medications, modeling patients’ course of care, and many other purposes. While more datasets of the scale of MIMIC are needed, they are achievable only through long-term effort. In the short term, significant first steps could be made for activity and participation information by developing and publishing an annotation schema for one or two specific aspects of activity, and by making a small set of annotated data available to the research community through existing data sharing mechanisms. This will enable rapid, effective communication in research via common reference points and shared benchmarking for evaluation.

### Action 2: Define analytic tasks

As a companion effort to developing these data resources and standards, we must also **identify and clearly define common research problems and applications for processing activity reports**. In computational research communities such as NLP, shared definitions of analytic tasks are the bones of effective research and evaluation. Identifying the characteristics of activity reports in structured and unstructured data and evaluating how these problems fit existing frameworks in NLP and other fields will enable development and adaptation of methods within the research community. Together with identifying downstream analytic tasks where information on activity and participation can be leveraged, such as cohort selection or rehospitalization risk prediction, this process will also help identify relevant data needs in collecting and storing activity reports. This task is thus interdependent with documentation and annotation standards; the challenge for analysis is to define how the information is to be automatically extracted and used. These problems and tasks must be defined with input from clinicians and data scientists alike. A major first step in this direction could be to develop a shared task for extracting one particular type of activity report from an annotated dataset. Such efforts promote broader research by laying the groundwork for the collaborative effort in developing and evaluating analytic methods.

### Action 3: Develop machine-readable ontologies

For both capturing and analyzing activity reports, it is critical to **develop a robust ontology that describes the components of activity and participation information and their relationships to one another and to other biomedical, psychological, and social concepts**. Such an effort has two major components: formalizing the conceptual framework and developing machine-readable resources. The first component involves defining the concepts necessary to represent activity and participation and activity reports, and capturing the necessary relationships between these concepts to describe their interaction. Many such resources and conceptual models—such as the ICF—already exist in rehabilitation medicine, mental health research, etc., and drawing on and connecting these proven resources should be the starting point for any analytically-focused effort. In addition, some important elements of activity and participation have coverage in other biomedical vocabularies such as SNOMED CT and LOINC; by mapping to these resources, well-developed analytic methods for clinical information can inform work on analyzing activity reports. As such models and mappings are developed, machine-readable implementations, similar to the UMLS, will enable analytic methods to build directly on the conceptual structure. One initial step towards this goal could be leveraging previous findings of activity and participation information in SNOMED [102] to develop mappings from SNOMED concepts to the ICF framework, providing a powerful tool for identifying and analyzing components of activity information. Development of ontological models needs to be a clinically-motivated process that is verified empirically, and thus must be developed in concert with engaged practitioners and researchers. Such standardized resources will support training in documenting activity and participation, as well as methods for analyzing it.

### Action 4: Establish documentation standards

A key step in improving the availability of information on activity and participation within healthcare delivery is to **establish standards for how and when to document activity and participation status during clinical encounters**. While this is a much larger task than a single paper can accomplish, potentially involving the coordinated efforts of international entities, multiple such standards have already been developed within rehabilitation medicine, as mentioned in the previous section; additionally, the Institute of Medicine has made some specific recommendations for documenting social and behavioral information in EHRs, including some activity and participation information [108]. However, awareness and adoption of these standards by the broader medical community are limited, and different standards compete even within the rehabilitation community. Establishing a single standard for the medical field at large to use is a long-term effort, but in the short term, small, focused efforts can be made within local institutions or health systems to increase the availability of activity reports. In some cases, such as team settings involving an occupational or physical therapist, activity reports are likely already being captured, and need only be intentionally analyzed. In other settings, relatively minimal interventions can capture high-impact activity and participation status. For example, a clinician could regularly note a patient’s ability to move through the clinic independently, and ask the patient if they are currently experiencing any limitations in their regular activities. Developing small sets of such practices can significantly improve the availability of activity reports within health records while broader standards are established.

## Conclusion

Function is an important indicator of health from both population and individual perspectives. However, information on function, and particularly on activity and participation, has not been used in a routine and standardized way when evaluating and monitoring the health of individuals from a holistic viewpoint. We believe rapid advances in data management and analytic tools have the potential to address barriers facing the effective use of activity and participation information, by locating, extracting, organizing, and summarizing activity reports from massive quantities of medical records. We find health informatics, and natural language processing in particular, to be a promising avenue for accelerating these efforts. Informatics can enable identification, extraction, and organization of activity and participation information for applications such as disability assessment and health monitoring [90, 91], and can also be used in software or devices to assist people with disabilities to engage in daily activities effectively [109, 110]. While existing applications of informatics methodologies to activity and participation information have shown promise, they face several challenges, including reliance on manual collection of non-standardized terminologies in text by domain experts, a lack of a shared systematic framework for activity and participation analysis, and a lack of relevant data. To drive informatics forward as a tool for capturing and utilizing activity and participation information, we recommend four important steps: (1) make activity and participation annotation standards and datasets available to the broader research community; (2) define common research problems in automatically processing activity and participation information; (3) develop robust, machine-readable ontologies for function that describe the components of activity and participation information and their relationships; and (4) establish standards for how and when to document activity and participation status during clinical encounters. These are challenging steps, requiring international coordination, but we provide short-term goals for each that can be accomplished in a reasonable timeframe and measurably improve ability to capture and use activity and participation data.

Whole-person function, as embodied by activity and participation, is a strong predictor of mortality, disability, employment, and resource utilization. Moreover, it outperforms comorbidities in predicting acute care readmissions in medically complex patients. We envision that standardized and accessible activity and participation information yielded from these efforts will provide valuable evidence-based knowledge that can be translated into practice by helping provide holistic and patient-centered care and ultimately improving the efficiency and effectiveness of health care delivery, management, and planning.

## Data Availability

Not applicable

## Abbreviations

ADLs: Activities of daily living
CMS: Centers for Medicare and Medicaid Services
DALYs: Disability adjusted life years
EHR: Electronic health record
ICD: International Classification of Diseases
ICF: International Classification of Functioning, Disability, and Health
ICIDH: International Classification of Impairments, Disabilities, and Handicaps
IRB: Institutional Review Board
LOINC: Logical Observation Identifiers Names and Codes
NLP: Natural language processing
PHI: Protected health information
SNOMED CT: SNOMED Clinical Terms
SNOMED: Systematized Nomenclature of Medicine
UMLS: Unified Medical Language System
UN: United Nations
WHO: World Health Organization
